# Metformin restores the mitochondrial membrane potentials in association with a reduction in TIMM23 and NDUFS3 in MPP+-induced neurotoxicity in SH-SY5Y cells

**DOI:** 10.17179/excli2019-1703

**Published:** 2019-09-10

**Authors:** Pitak Chanthammachat, Permphan Dharmasaroja

**Affiliations:** 1 Department of Anatomy, Faculty of Science, Mahidol University, Bangkok 10400, Thailand

**Keywords:** MPP+, metformin, mitochondria, SH-SY5Y cells, Parkinson's disease

## Abstract

SH-SY5Y cells exposed to 1-methyl-4-phenylpyridinium (MPP^+^) develop mitochondrial dysfunction and other cellular responses similar to those that occur in the dopaminergic neurons of patients with Parkinson's disease (PD). It has been shown in animal models of PD that neuronal death can be prevented by metformin, an anti-diabetic drug. Both MPP^+^ and metformin inhibit complex I of the mitochondrial respiratory chain. It has been reported that decreased levels of the mitochondrial inner membrane proteins TIMM23 and NDUFS3 are associated with the increased generation of reactive oxygen species and mitochondrial depolarization. In the present study, we investigated the effects of metformin on MPP^+^-induced neurotoxicity using differentiated human SH-SY5Y neuroblastoma cells. The results showed that pretreatment with metformin increased the viability of MPP^+^-treated SH-SY5Y cells. Pretreatment with metformin decreased the expression of TIMM23 and NDUFS3 in MPP^+^-treated SH-SY5Y cells. This was correlated with reduced mitochondrial fragmentation and an improvement in the mitochondrial membrane potential. These results suggest that metformin pretreatment protects against MPP^+^-induced neurotoxicity, and offer insights into the potential role of metformin in protecting against toxin-induced parkinsonism.

## Introduction

l-methyl-4-phenylpyridinium (MPP^+^) is the active metabolite of the neurotoxin 1-methyl-4-phenyl-l,2,3,6-tetrahydropyridine (MPTP) and can block the transportation of electrons by inhibiting complex I of mitochondria, leading to a loss of ATP production (Cassarino et al., 1999[2]). MPP^+^ can also open the mitochondrial transition pore and cause the release of Ca^2+^ and cytochrome *c* by means of an oxidative mechanism, which can lead to apoptotic cell death. MPTP is a contaminant that is produced during the illicit synthesis of 1-methyl-4-phenyl-4-propionoxypiperidine (MPPP), an analog of narcotic meperidine (Dauer and Przedborski, 2003[4]). Once MPTP enters into the blood, it rapidly diffuses through the blood-brain barrier into the brain and is immediately converted to N-methyl-4-phenyl-2,3-dihydropyridinium (MPDP^+^) in the outer mitochondrial membrane of the nondopaminergic neurons, where the MPDP^+^ is spontaneously oxidized to MPP^+^. Then, MPP^+^ enters the dopaminergic neurons through the dopamine transporter and accumulates in the inner mitochondrial membrane, where it inhibits complex I, causing the parkinsonian behavior in humans and rodents (Meredith and Rademacher, 2011[24]).

Recently, two studies in an MPTP-induced mouse model of Parkinson's disease showed that dopaminergic neuron death can be prevented by the administration of metformin (Patil et al., 2014[25]; Lu et al., 2016[21]). Metformin is a drug used for the treatment of type 2 diabetes due to its ability to decrease hepatic glucose production, which leads to the activation of AMP-activated protein kinase (AMPK) (Zhou et al., 2001[37]). At present, metformin is widely accepted as an AMPK activator that accelerates AMPK phosphorylation and induces macroautophagy and mitophagy (Xie et al., 2011[36]; Kang et al., 2016[14]). The mechanism of the neuroprotective effect of metformin has been investigated in MPP^+^-treated SH-SY5Y neuroblastoma cells, and it has been shown that metformin can activate AMPK in SH-SY5Y cells and in turn induce microtubule-associated protein 1 light chain 3-II (LC3-II)-mediated autophagy and mitochondrial reactive oxygen species (ROS) clearance (Lu et al., 2016[21]). 

The role of metformin, as an AMPK activator (Lu et al., 2016[21]) and mTOR inhibitor (Perez-Revuelta et al., 2014[26]), in protecting against neuronal death in models of PD remains controversial. Studies in both wild-type AMPK and AMPK knockout mice have suggested that the neuroprotective effects of metformin are not due to AMPK activation in dopaminergic neurons (Bayliss et al., 2016[1]). AMPK controls energy metabolism by regulating mitochondrial ATP synthesis and consumption (Hardie et al., 2012[10]; Ke et al., 2018[16]).

A breakthrough study showed that metformin selectively inhibits complex I (NADH:ubiquinone oxidoreductase) of the mitochondrial respiratory chain and, as a result, decreases NADH oxidation, reduces the proton gradient across the inner mitochondrial membrane, and reduces oxygen consumption rate (El-Mir et al., 2000[7]). Chlorpyrifos treatment in SH-SY5Y cells induces apoptosis and decreases the levels of the mitochondrial inner membrane proteins TIMM23 (Translocase of Inner Mitochondrial Membrane 23) and NDUFS3 (NADH Dehydrogenase (Ubiquinone) Fe-S Protein 3) in addition to increasing ROS generation and mitochondrial depolarization (Dai et al., 2015[3]). TIMM23 is an essential component of the TIMM23 complex, a complex that mediates the translocation of transit peptide-containing proteins across the mitochondrial inner membrane (Demishtein-Zohary and Azem, 2017[5]). NDUFS3 is a core subunit of NADH dehydrogenase (complex I), and the cleavage of NDUFS3 triggers a programmed cell death pathway that leads to mitochondrial dysfunction and the generation of ROS (Lieberman, 2010[19]). A study in a pulmonary epithelial carcinoma cell line showed that cells that are deficient in NDUFS3 exhibit increased resistance to metformin, resulting in increased tumor growth (Wheaton et al., 2014[34]). There is no data on whether metformin plays a role in the expression of TIMM23 and NDUFS3 in neuronal cells and thus in toxin-induced neuronal cell death.

In the present study, the SH-SY5Y cell line was chosen as a cellular model to investigate the potential protective effect of metformin against MPP^+^-induced neuronal cell death. MPP^+^-exposed SH-SY5Y cells, after being differentiated with retinoic acid into tyrosine hydroxylase-expressing neuronal cells (Kovalevich and Langford, 2013[18]), are widely used as a PD model (Xicoy et al., 2017[35]). Here, we investigated the effects of metformin on cell viability, the protein expression of the mitochondrial inner membrane proteins TIMM23 and NDUFS3, mitochondrial morphology and the mitochondrial membrane potential.

## Materials and Methods

### Materials

MTT (3-(4,5-dimethylthiazol-2-yl)-2,5-diphenyl-2H-tetrazolium bromide), protease inhibitor cocktail, an LC-3B primary antibody, metformin hydrochloride, and other chemicals were purchased from Sigma-Aldrich (St. Louis, MO, USA). Primary antibodies against TIMM23, and NDUFS3 as well as a JC-10 Mitochondrial Membrane Potential Assay Kit were purchased from Abcam (Cambridge, UK). Secondary antibodies conjugated to horseradish peroxidase (HRP) were purchased from Cell Signaling Technology (Danvers, MA, USA). Bradford Protein Assay Reagent was purchased from Bio-Rad Laboratories (Hercules, CA, USA). Enhanced Chemiluminescence (ECL) Western blotting Substrate was purchased from Pierce Biotechnology (Rockford, IL, USA). An RNA extraction kit, High Capacity Reverse Transcription Kit, and Kapa SYBR^®^ FAST qPCR Kit (ABI Prism) were purchased from Qiagen (Germantown, MD, USA), Applied BioSystems (Foster City, CA, USA) and Kapa Biosystems (Sigma-Aldrich, St. Louis, MO, USA), respectively.

### Cell culture and treatment

Human neuroblastoma SH-SY5Y cells were cultured in a 1:1 mixture of Dulbecco's modified Eagle medium (DMEM) and Nutrient Mixture Ham's F-12 medium supplemented with 10 % heat-inactivated fetal bovine serum (FBS), 1 mM sodium pyruvate, and 0.1 M nonessential amino acid. Cells were propagated in a 25-cm^2^ T-flask in a 95 % humidified air incubator at 37 ºC and in 5 % CO_2_. In the experiment, the cells were subcultured with 0.25 % porcine pancreatic trypsin and then plated into sterile culture flasks or culture plates, depending on the experimental design. The cells were incubated for 2 d prior to treatment with all-*trans* retinoic acid (RA), MPP^+^, or metformin, depending on the experimental design. For the neuronal differentiation of SH-SY5Y cells, RA was added at the final concentration of 50 μM in MEM-F12 with 1 % FBS and maintained for 10 d.

### Cell viability assay

Cell viability was measured by using the MTT assay. Basically, the tetrazolium ring in MTT is reduced to purple formazan by mitochondrial dehydrogenases with NADH in active mitochondria. SH-SY5Y cells were seeded in a 96-well culture plate at a density of 6 x 10^2 ^cells/well in 200 µl of culture medium and incubated at 37 ºC in 5 % CO_2_ in a humidified incubator for 2 d. A total of 5 mg/ml MTT solution was then added to the culture medium, and the cells were incubated for 4 h at 37 ºC in a humidified atmosphere containing 5 % CO_2_. Then, the medium was removed and replaced with MTT solubilization solution to allow the dissolution of formazan crystals. The absorbance of the samples was measured at 570 nm and normalized to the absorbance at wavelength 690 nm using a Biotek Synergy HT microplate reader with KC4 software. 

### Western blot analysis

SH-SY5Y cells were plated in 6-well plates at a density of 2 x 10^4^ cells/well and incubated overnight. After differentiation and treatment, the conditioned medium was discarded, and the cells were washed three times with cold PBS. Then, the cells were lysed in RIPA lysis buffer containing a protease inhibitor cocktail for 10 min at 4 ºC or on ice. Then, cells were scraped off, and the lysates were collected. The extracted lysates were centrifuged at 14,000 rpm for 15 min at 4 ºC, and the supernatants were collected and stored at -80 ºC, or immediately used to determine the protein concentration by the Bradford method. An equal amount (30 μg) of protein from each experimental group was separated by 10-15 % SDS-polyacrylamide gel electrophoresis. After that, the proteins on the gel were transferred to a nitrocellulose membrane using Towbin's buffer with 20 % methanol for low molecular weight proteins or with 10 % methanol plus 0.05 % SDS for high molecular weight proteins at 100 V for 1 h and 45 min. The nonspecific binding was blocked by 5 % skimmed milk for 1 h at room temperature, and the membrane was washed three times for 10 min each with TBST buffer and sequentially incubated with a 1:1000 dilution of a TIMM23, or NDUFS3 primary antibody, or a 1:5000 dilution of a monoclonal β-actin primary antibody overnight at 4 ºC. After washing, the membrane was then incubated with a 1:20000 dilution of HRP-conjugated goat anti-rabbit IgG as the secondary antibody for target proteins or a 1:5000 dilution of HRP-conjugated horse anti-mouse IgG (Invitrogen, Eugene, OR, USA) as the secondary antibody for β-actin at room temperature for 1 h. For tyrosine hydroxylase (TH), the membrane was incubated with a primary rabbit polyclonal antibody against TH (1:1000 dilution; Cell Signal Technology) overnight. Subsequently, the membrane was incubated with an HRP-conjugated goat anti-rabbit secondary antibody (1:5000 dilution) for 45 min at room temperature. The protein bands were visualized using ECL Prime Western Blotting Substrate. Finally, the blots were scanned, and densitometry analysis was performed on the scanned images using ImageJ software (National Institutes of Health, MD, USA).

### Immunostaining for tyrosine hydroxylase 

SH-SY5Y cells were plated on poly-L-lysine-coated coverslips at a density of 1 x 10^4^ cells/coverslip. The cells were treated with 10 μM of RA for 5 and 10 d. The cells were then fixed in 4 % paraformaldehyde for 15 min at room temperature followed by sequential incubation with permeabilizing solution (0.2 % Triton X-100) in PBS for 30 min at room temperature. Then, the cultures were washed again with PBS and incubated in blocking solution (3 % BSA in 0.5 % Tween 20 in PBS) for 30 min. The cells were incubated with a rabbit polyclonal antibody against TH (1:200 dilution in blocking solution; Merck Millipore, MA, USA) overnight at 4 ºC. After washing, the cells were incubated with a 1:500 dilution of an Alexa 488-conjugated secondary antibody for 1 h at room temperature. The coverslips were then mounted with Vectashield Antifading Mounting Medium (Vector Laboratories, CA, USA). The cells were visualized under a laser scanning confocal microscope (Olympus Model FV 1000, Tokyo, Japan).

### Immunostaining for TIMM23 and NDUFS3

SH-SY5Y cells were plated on poly-L-lysine coated coverslips in 24-well culture plates at a density of 4 x 10^3^ cells/well and incubated at 37 ºC in 5 % CO_2_ in a humidified incubator for 24 h. After treatment according to the experimental design, the cells were rinsed with PBS, fixed with 4 % paraformaldehyde in PBS (pH 7.4), and washed with PBS. Nonspecific binding was blocked with 5 % normal goat serum. The cells were incubated with a 1:200 dilution of a TIMM23 and NDUFS3 primary antibody overnight at 4 ºC. After washing, the cells were incubated with a 1:500 dilution of an Alexa-594 conjugated secondary antibody for 1 h at room temperature. For mitochondrial staining, the cells were incubated in medium with MitoTracker red-labeled probes for 30 min at 37 ºC, and then washed. Finally, the nuclei were stained with DAPI in antifade mounting medium. Images were obtained using a laser scanning confocal microscope (FV10i-DOC; Olympus Life Science, MA, USA).

### Mitochondrial membrane potential assay

SH-SY5Y cells were seeded in 6-well plates at a density of 2 x 10^4^ cells/well in 2 ml of medium and then incubated at 37 ºC in 5 % CO_2_ in a humidified incubator for 24 h. After treatment according to the experimental design, the cells were trypsinized and centrifuged at 300 rpm for 5 min. The pellet was gently washed twice with cold PBS and resuspended in 500 µl of JC-10 dye solution at a density of 3 x 10^5^ cells/tube and incubated at 37 ºC in 5 % CO_2_ for 30 min (in the dark). The membrane potential was analyzed with a flow cytometer by measuring the fluorescence with excitation at 488 nm and emission at 530 nm and 585 nm.

### Statistical analysis

All data were expressed as the means ± SD or SEM of at least three independent experiments. Differences between the control and treatment groups were evaluated by one-way ANOVA followed by Turkey's post hoc test using GraphPad Prism version 5.0 software (GraphPad Software, San Diego, CA, USA). A *P*-value < 0.05 was considered statistically significant.

## Results

### Differentiation of SH-SY5Y cells

**Figure 1:**
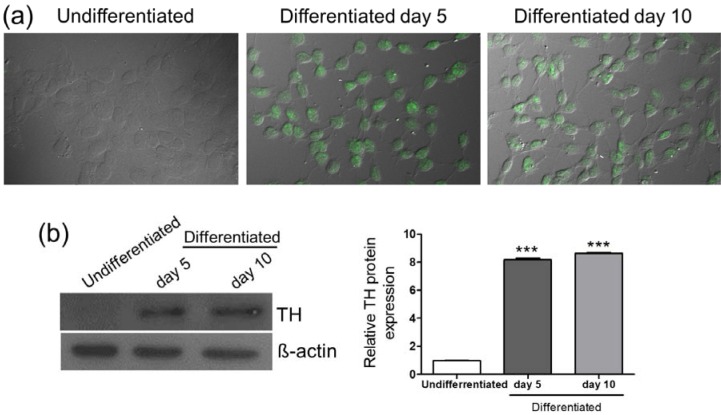
RA-induced differentiation of SH-SY5Y cells. Cells were treated with RA (50 µM) for 5 and 10 d. (a) After RA treatment, the expression of tyrosine hydroxylase (TH) was visualized by immunostaining in undifferentiated cells and cells differentiated with RA for 5 or 10 d. (b) TH protein was detected by Western blotting. β-actin was used as a normalization control. The density of bands was analyzed based on the expression of β-actin. Data are expressed as mean ± SD from three independent experiments. *** *P *< 0.001 versus the undifferentiated group

To examine the effects of MPP^+^ and metformin on neuronal cells, SH-SY5Y neuroblastoma cells were differentiated with 50 µM RA for 10 d. The RA-treated cells were then stained with the dopaminergic neuronal marker tyrosine hydroxylase (TH) and observed under a confocal microscope. The RA-treated cells showed increased expression of TH on day 5 and day 10, and morphological changes in neuronal-like cells, including the elongation of the neurites and neural network formation were observed, especially on day 10 (Figure 1a(Fig. 1)). In addition, compared with that in the undifferentiated cells, the protein expression of TH was significantly increased in the RA-differentiated cells on day 5 and day 10 (Figure 1b(Fig. 1)). These results suggested that SH-SY5Y cells were differentiated into neuronal-like cells with a dopaminergic phenotype upon RA treatment, and this differentiation protocol was used for subsequent experiments in this study.

### Metformin increased the viability of MPP^+^-treated differentiated SH-SY5Y cells

**Figure 2:**
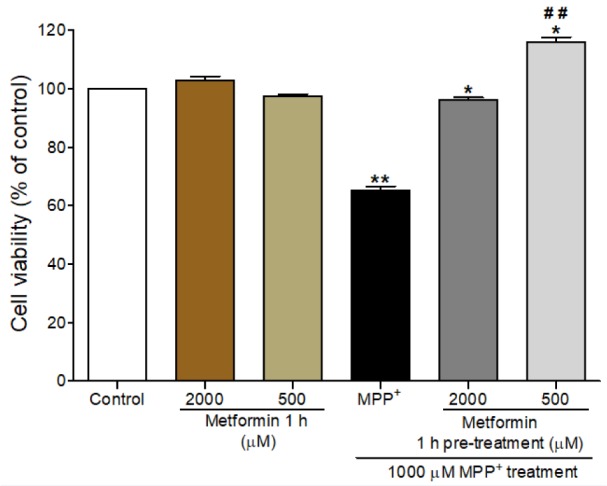
Effects of metformin pretreatment on the viability of MPP^+^-treated differentiated SH-SY5Y cells. Cell viability was determined by the MTT assay. Cells were pretreated with metformin (2000 µM and 500 µM) for 1 h before exposure to MPP^+^ (1000 µM) for 24 h.The data are expressed as the means ± SEM from five independent experiments, each of which included five replicates. *** *P* < 0.001 versus control; ### *P* < 0.001 versus MPP^+^-treated cells

To determine the suitable concentration of metformin, we assessed the effect of metformin and MPP^+^ on the viability of differentiated SH-SY5Y cells by using an MTT assay. The differentiated cells were pretreated with 500 µM or 2000 µM of metformin for 1 h prior to exposure to 1000 µM MPP^+^ for 24 h. Exposure to MPP^+^ alone induced a significant reduction in cell viability (65.22 % ± 1.4; *P* < 0.001) compared to that in the control, while treatment with 500 µM or 2000 µM of metformin alone did not affect the cell viability (Figure 2(Fig. 2)). Pretreatment with 500 µM or 2000 µM of metformin for 1 h followed by a 24 h of exposure to 1000 µM MPP^+^ significantly increased the viability of differentiated SH-SY5Y cells (*P* < 0.001). A metformin concentration of 500 µM of metformin was chosen for subsequent experiments.

### Metformin induced a reduction in the mitochondrial proteins TIMM23 and NDUFS3 in MPP^+^-treated SH-Y5Y cells

**Figure 3:**
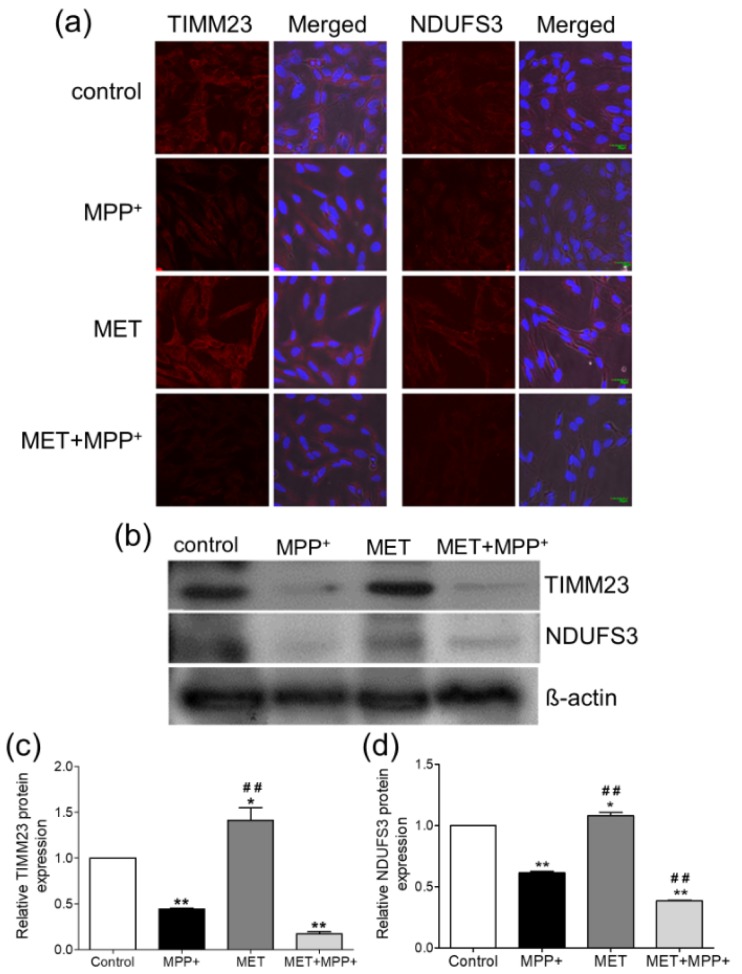
Effects of metformin pretreatment on the expression of the mitochondrial proteins TIMM23 and NDUFS3 in MPP^+^-treated differentiated SH-SY5Y cells. Cells were pretreated with metformin (500 µM) for 1 h before exposure to MPP^+^ (1000 µM) for 24 h. (a) Immunostaining for TIMM23 and NDUFS3. The nuclei were stained with DAPI. (b) Protein expression was examined by Western blotting. ß-actin was used as a normalization control. (c-d) The bar graphs show the quantifications of TIMM23 and NDUFS3 expression. The data are expressed as the means ± SD from three independent experiments. * *P* < 0.05, ** *P* < 0.01, *** *P* < 0.001 versus control; ### *P* < 0.001 versus MPP^+^-treated cells

Differentiated SH-SY5Y cells treated with 1000 µM MPP^+^ for 24 h exhibited a reduction in the fluorescent signals of TIMM23 and NDUFS3, while cells treated with 500 µM metformin for 1 h exhibited higher fluorescent signals (Figure 3a(Fig. 3)). Pretreatment with metformin prior to MPP^+^ resulted in decreases in the fluorescent signals of TIMM23 and NDUFS3. These results were consistent with the densitometric analysis of the immunoblots (Figure 3b(Fig. 3)). The MPP^+^-treated cells showed significant decreases in the expression levels of TIMM23 (-1.56-fold change; *P* < 0.01; Figure 3c(Fig. 3)) and NDUFS3 (-1.39-fold change; *P* < 0.001; Figure 3d(Fig. 3)). Treatment with metformin alone significantly increased the expression of TIMM23 (1.41-fold; *P* < 0.05; Figure 3c(Fig. 3)) and NDUFS3 (1.08-fold; *P* < 0.05; Figure 3d(Fig. 3)) when compared to that in the control. However, when compared to MPP^+^ treatment only, pretreatment with metformin prior to MPP^+^ exposure induced a relative decrease in the expression of TIMM23 (-1.83-fold change; *P* <0.001; Figure 3c(Fig. 3)) and a significant decrease in NDUFS3 expression (-1.63-fold change; *P* < 0.001; Figure 3d(Fig. 3)).

### Metformin protected the mitochondrial networks and reversed the membrane potential in MPP^+^-treated SH-SY5Y cells

**Figure 4:**
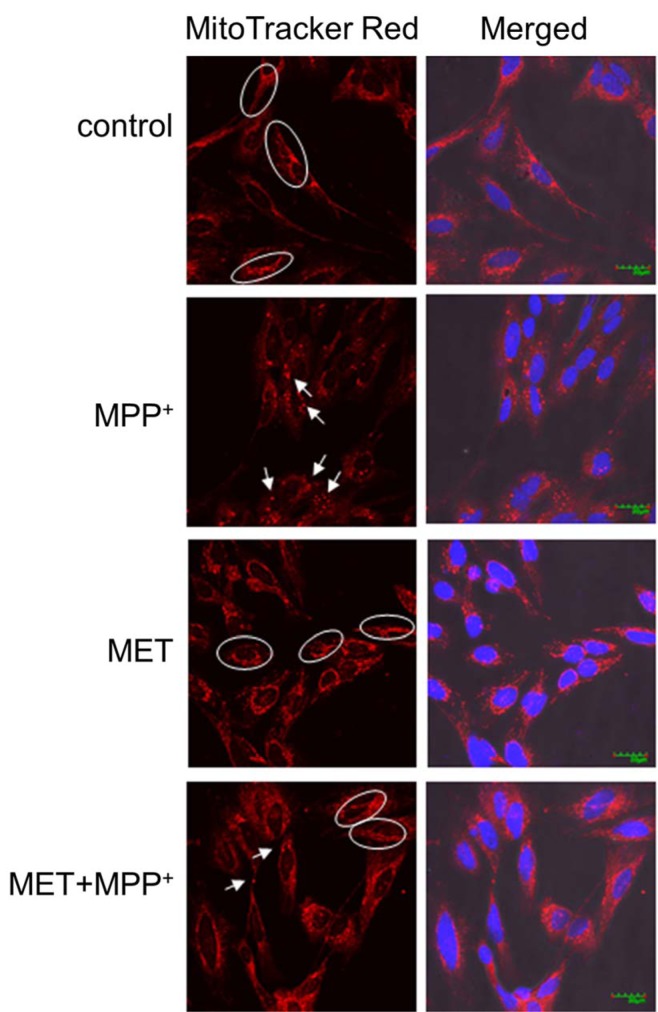
Metformin protected mitochondrial networks in MPP^+^-treated differentiated SH-SY5Y cells. Cells were pretreated with metformin (500 µM) for 1 h before exposure to MPP^+^ (1000 µM) for 24 h. The mitochondria were labeled with MitoTracker red-labeled probes, and the nuclei were stained with DAPI. Images were obtained using a laser scanning confocal microscope. The circled areas indicate a tubular or thread-like appearance of the mitochondria, and the arrows indicate short-rod or sphere-like appearance.

To determine whether the expression of TIMM23 and NDUFS3 was related to the changes in mitochondrial morphology induced by metformin in MPP^+^-treated SH-SY5Y cells, MitoTracker dyes were used for mitochondrial labeling. Mitochondria in the control cells were filamentous with a tubular or thread-like appearance and often formed an interconnected network (Figure 4(Fig. 4)). After exposure to 1000 µM MPP^+^ for 24 h, the mitochondrial networks in the SH-SY5Y cells were fragmented into short rods or spheres. Treatment with 500 µM metformin for 1 h alone did not affect the mitochondrial networks. Interestingly, pretreatment with metformin prior to MPP^+^ exposure protected the mitochondrial networks from being fragmented by MPP^+^ as shown by the preservation of tubular or thread-like appearances.

**Figure 5:**
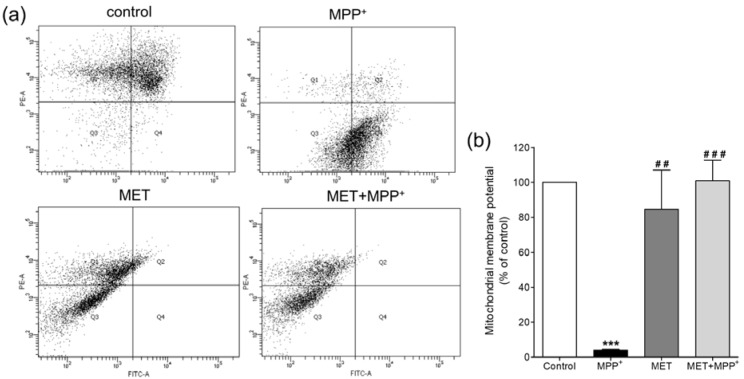
Effects of metformin pretreatment on the mitochondrial membrane potential in MPP^+^-treated differentiated SH-SY5Y cells. Cells were pretreated with metformin (500 µM) for 1 h before exposure to MPP^+^ (1000 µM) for 24 h. The mitochondrial membrane potential was examined using the fluorescent JC-10 probe and analyzed with a flow cytometer. (a) The scatter plots show the quantity of cells emitting light of JC-10 reversible dye. Q1 indicates the quantity of cells emitting light of JC-10 in its monomer form, and Q4 indicates the quantity of cells emitting light of JC-10 in its aggregated form. (b) The bar graphs show the quantification of cells emitting light of JC-10 in the monomer form as a percentage of the control. The data are expressed as the means ± SD from three independent experiments. *** *P* < 0.001 versus control; ## *P* < 0.01, ### *P* < 0.001 versus MPP^+^-treated cells

As the inner membrane of mitochondria helps maintain the transmembrane gradient of ions, the membrane potential of mitochondria was further investigated to determine the effect of metformin in MPP^+^-treated SH-SY5Y cells. The mitochondrial membrane potential was monitored using a fluorescent JC-10 probe, which passes through the mitochondrial membrane and accumulates in the mitochondrial matrix and the accumulation of which is maintained by the membrane potential (ΔΨm). Flow cytometry analysis of the JC-10 probe showed that SH-SY5Y cells exposed to 1000 µM MPP^+^ for 24 h exhibited a significant decrease in the mitochondrial membrane potential (*P *< 0.001) (Figure 5(Fig. 5)). Treatment with 500 µM metformin alone did not affect the membrane potential compared to that in the control. However, pretreatment with metformin prior to MPP^+^ exposure helped maintain the mitochondrial membrane potential at a level approximately equal to that of the control. This suggested that pretreatment with metformin protected the membrane potential changes caused by MPP^+^.

For more results see the Supplementary data.

## Discussion

The MPP^+^-induced death of dopaminergic neurons is mediated via mitochondrial dysfunction. Evidence has suggested that MPP^+^ can induce autophagy, halt mitochondrial trafficking, and the ΔΨm (Kim-Han et al., 2011[17]; Hung et al., 2014[11]). The present study showed that pretreatment with metformin protected against the MPP^+^-induced death of differentiated SH-SY5Y dopaminergic cells. As such, the results demonstrated that 1) metformin increased the viability of the MPP^+^-treated SH-SY5Y cells; 2) metformin increased the expression of TIMM23 and NDUFS3 in SH-SY5Y cells, although the expression of these proteins was reduced upon treatment MPP^+^; and 3) metformin protected against MPP^+^-induced mitochondrial fragmentation and changes in the ΔΨm. 

Although the results of the cell viability assay are supported by previous studies, the mechanism by which metformin protects against MPP^+^-induced neuronal loss remains unclear. Our results showed that metformin pretreatment prior to MPP^+^ exposure decreased the levels of the mitochondrial inner membrane proteins TIMM23 and NDUFS3 in SH-SY5Y cells. A previous study suggested that a reduction in these proteins indicates enhanced mitophagy (Hung et al., 2014[11]). A recent study suggested that metformin can activate AMPK in MPP^+^-treated SH-SY5Y cells and in turn induce microtubule-associated protein 1 light chain 3-II (LC3-II)-mediated autophagy and mitochondrial ROS clearance (Lu et al., 2016[21]). Similar findings have been observed in organophosphate-induced apoptosis in SH-SY5Y cells (Dai et al., 2015[3]). Substantial evidence has shown that mitophagy plays a protective role under various pathological conditions by removing damaged mitochondria (Jin et al., 2010[13]; Meissner et al., 2011[23]). Our results showed that, in association with a reduction in TIMM23 and NDUFS3 expression, metformin also reduced mitochondrial fragmentation in SH-SY5Y cells and maintained the ΔΨm at approximately normal levels. Previous studies in other diseases, such as Down syndrome and diabetes, have also indicated the role of metformin in reducing mitochondrial fragmentation and restoring the ΔΨm (Izzo et al., 2017[12]; Wang et al., 2017[33]; Ma et al., 2019[22]). 

The results of the present study showed that metformin alone increased the TIMM23 and NDUFS3 protein expression. The proteins are involved in the formation of a ΔΨ-dependent protein-conducting pore across the inner membrane, which allows the translocation of the matrix proteins and intermembrane space proteins (Truscott et al., 2001[29]; Suhane et al., 2013[28]). It was recently shown that TIMM23 overexpression partially protects against MPP^+^-induced cell death in the human neuroblastoma dopaminergic cell line BE(2)-M17 (Franco-Iborra et al., 2018[8]). From our results, it can be assumed that metformin pretreatment upregulates the expression of TIMM23 and NDUFS3 to levels that are more than sufficient for MPP^+^-induced mitophagy and to maintain the mitochondrial network and the ΔΨm, allowing for cell survival.

A potential limitation of our study is that we did not directly measure markers of autophagy such as LC3, p62, Atg5 or Beclin 1. In particular, for mitophagy, the localization of the autophagosomal marker MAP1LC3B in mitochondria needs to be investigated (Dolman et al., 2013[6]). As the loss of ΔΨm is a trigger for mitophagy (Twig and Shirihai 2011[30]), we use JC-10, a potentiometric probe and a derivative of JC-1 commonly used to study the ΔΨm, to reflect mitophagy (Gegg et al., 2009[9]).

Three animal studies have demonstrated the ability of metformin to protect against dopaminergic neuronal death induced by the neurotoxin MPTP, which correlates with improvements in motor function (Patil et al., 2014[25]; Lu et al., 2016[21]; Katila et al., 2017[15]). Given that both MPTP and metformin act on complex I of the respiratory chain, resulting in a decrease in the ΔΨm (El-Mir et al., 2000[7]), a mutual influence of the drugs on mitochondrial survival cannot be excluded. It is possible that, in these studies, metformin primarily reduces the damaging effects of MPTP itself rather than restoring damaged neurons (Rotermund et al., 2018[27]). In cellular models of toxin-induced toxicity, metformin possibly exerts different effects on mitochondria depending on cell type, and the concentrations of the toxin, and the duration of exposure to the toxin (Vial et al., 2019[31]). The exposure of LNCaP prostate cancer cells to 5 mM of metformin for 24 h causes mitochondrial swelling but does not induce the fission of the mitochondrial network (Loubiere et al., 2017[20]). In undifferentiated SH-SY5Y neuroblastoma cells, pretreatment with 2 mM metformin for 1 h reverse ΔΨm changes induced by 48-h exposure to 200 µM MPP^+^ (Lu et al., 2016[21]). Our results showed that pretreatment with 500 µM of metformin for 1 h in differentiated SH-SY5Y cells prevented impairments in the ΔΨm induced by 1000 µM MPP^+^ for 24 h. 

Collectively, the present study showed that metformin pretreatment protects against MPP^+^-induced neurotoxicity, and this effect is associated with a reduction in the mitochondrial inner membrane proteins, suggesting mitophagy, and the maintenance of mitochondrial networks and the membrane potential. Supposedly, the mitophagic process helps with the degradation of dysfunctional mitochondria, while lesser-damaged mitochondria remain functional and support cell survival. An epidemiological study suggested that metformin can substantially reduce the risk of Parkinson's disease in diabetes (Wahlqvist et al., 2012[32]). Our findings may explain the positive effect of metformin in this particular group of patients and may suggest that it has beneficial effects in toxin-induced parkinsonism.

## Acknowledgements

This research is under the research framework of Mahidol University. This work was supported by Department of Anatomy, Faculty of Science, Mahidol University.

## Conflict of interest

The authors declare no conflict of interest.

## Supplementary Material

Supplementary data
