# Supplementary material for: Metformin restores the mitochondrial membrane potentials in association with a reduction in TIMM23 and NDUFS3 in MPP+-induced neurotoxicity in SH-SY5Y cells
Source: EXCLI J. 2019 Sep 10;18:812–23. doi: 10.17179/excli2019-1703 (PMC6806136; doi:10.17179/excli2019-1703)
Supplement: Supplementary data [file EXCLI-18-812-s-001.pdf]

EXCLI Journal 2019;18:812-823 – ISSN 1611-2156
Received: August 05, 2019, accepted: September 03, 2019, published: September 10, 2019

Supplementary material to:

METFORMIN RESTORES THE MITOCHONDRIAL MEMBRANE POTENTIALS IN ASSOCIATION WITH A REDUCTION IN TIMM23 AND NDUFS3 IN MPP$^+$-INDUCED NEUROTOXICITY IN SH-SY5Y CELLS

Pitak Chanthammachat, Permphan Dharmasaroja*

Department of Anatomy, Faculty of Science, Mahidol University, Bangkok 10400, Thailand

* Corresponding author: Permphan Dharmasaroja, Department of Anatomy, Faculty of Science, Mahidol University, Bangkok 10400, Thailand, Telephone number: +66-2-2015447, Fax number: +66-2-3547168, E-mail: permphan.dha@mahidol.ac.th

http://dx.doi.org/10.17179/excli2019-1703

This is an Open Access article distributed under the terms of the Creative Commons Attribution License (http://creativecommons.org/licenses/by/4.0/).

Supplementary Table 1: Band intensity of the proteins measured by the ImageJ software

	TH			β-actin			TH/β-actin		
	n1	n2	n3	n1	n2	n3	n1	n2	n3
**Undiff**	1363	1483	1505	15011	15045	14868	0.0908	0.098571	0.101224
**Diff_D5**	12117	11292	11866	14869	14126	14166	0.8149	0.799377	0.837639
**Diff_D10**	13188	14282	14655	15529	16490	16843	0.8492	0.866101	0.870094

TH, tyrosine hydroxylase
Undiff, undifferentiated SH-SY5Y cells
Diff_D5, SH-SY5Y cells differentiated with retinoic acid for 5 days
Diff_D10, SH-SY5Y cells differentiated with retinoic acid for 10 days
n, number of experiment
(Raw data to Figure 1, main document)

EXCLI Journal 2019;18:812-823 – ISSN 1611-2156
Received: August 05, 2019, accepted: September 03, 2019, published: September 10, 2019

Supplementary Table 2: Cell viability of differentiated SH-SY5Y cells measured by the MTT assay

n1	Control	MPP+	MET only 2000 µm	MET only 500 µm	Pretreat MET 2000 µM	Pretreat MET 500 µM
Rep1	0.554	0.381	0.579	0.649	0.562	0.684
Rep2	0.56	0.367	0.696	0.65	0.534	0.655
Rep3	0.616	0.356	0.501	0.573	0.538	0.6
Rep4	0.539	0.393	0.558	0.638	0.57	0.661
Rep5	0.585	0.301	0.511	0.606	0.559	0.668
Mean	0.5708	0.3596	0.569	0.6232	0.5526	0.6536

n2	Control	MPP+	MET only 2000 µm	MET only 500 µm	Pretreat MET 2000 µM	Pretreat MET 500 µM
Rep1	0.585	0.382	0.589	0.564	0.587	0.645
Rep2	0.537	0.312	0.634	0.659	0.524	0.608
Rep3	0.52	0.34	0.561	0.575	0.557	0.695
Rep4	0.598	0.332	0.678	0.584	0.553	0.654
Rep5	0.564	0.349	0.592	0.616	0.547	0.638
Mean	0.5608	0.343	0.6108	0.5996	0.5536	0.648

n3	Control	MPP+	MET only 2000 µm	MET only 500 µm	Pretreat MET 2000 µM	Pretreat MET 500 µM
Rep1	0.596	0.348	0.538	0.536	0.537	0.607
Rep2	0.547	0.407	0.519	0.579	0.529	0.619
Rep3	0.541	0.337	0.557	0.58	0.528	0.597
Rep4	0.5	0.367	0.597	0.539	0.505	0.62
Rep5	0.576	0.375	0.569	0.59	0.545	0.646
Mean	0.552	0.3668	0.556	0.5648	0.5288	0.6178

n4	Control	MPP+	MET only 2000 µm	MET only 500 µm	Pretreat MET 2000 µM	Pretreat MET 500 µM
Rep1	0.576	0.319	0.398	0.515	0.543	0.631
Rep2	0.539	0.382	0.501	0.584	0.52	0.597
Rep3	0.585	0.373	0.55	0.556	0.592	0.629
Rep4	0.524	0.351	0.511	0.533	0.486	0.654
Rep5	0.516	0.394	0.358	0.598	0.493	0.635
Mean	0.548	0.3638	0.4636	0.5572	0.5268	0.6292

EXCLI Journal 2019;18:812-823 – ISSN 1611-2156
Received: August 05, 2019, accepted: September 03, 2019, published: September 10, 2019

Supplementary Table 2 (cont.): Cell viability of differentiated SH-SY5Y cells measured by the MTT assay

n5	Control	MPP^+	MET only 2000 µm	MET only 500 µm	Pretreat MET 2000 µM	Pretreat MET 500 µM
Rep1	0.522	0.379	0.597	0.497	0.44	0.605
Rep2	0.581	0.396	0.599	0.462	0.518	0.651
Rep3	0.531	0.373	0.616	0.355	0.531	0.684
Rep4	0.545	0.361	0.612	0.482	0.549	0.691
Rep5	0.524	0.359	0.589	0.495	0.5	0.683
Mean	0.5406	0.3736	0.6026	0.4582	0.5076	0.6628

Mean of Mean	Control	MPP^+	MET only 2000 µm	MET only 500 µm	Pretreat MET 2000 µM	Pretreat MET 500 µM
n1	0.5708	0.3596	0.569	0.6232	0.5526	0.6536
n2	0.5608	0.343	0.6108	0.5996	0.5536	0.648
n3	0.552	0.3668	0.556	0.5648	0.5288	0.6178
n4	0.548	0.3638	0.4636	0.5572	0.5268	0.6292
n5	0.5406	0.3736	0.6026	0.4582	0.5076	0.6628
Mean	0.55444	0.36136	0.5604	0.5606	0.53388	0.64228

%Control	Control	MPP^+	MET only 2000 µm	MET only 500 µm	Pretreat MET 2000 µM	Pretreat MET 500 µM
n1	100	62.9993	99.6847	109.1801	96.8115	114.5060
n2	100	61.1626	108.9158	106.9187	98.7161	115.5492
n3	100	66.4493	100.7246	102.3188	95.7971	111.9203
n4	100	66.3869	84.5985	101.6788	96.1314	114.8175
n5	100	69.1084	111.4687	84.7577	93.8957	122.6045
Mean	100	65.2213	101.0785	100.9708	96.2704	115.8795

MPP^+, 1-methyl-4-phenylpyridinium
MET, metformin
n, number of experiment
rep, replicate number
(Raw data to Figure 2, main document)

Received: August 05, 2019, accepted: September 03, 2019, published: September 10, 2019

Supplementary Table 3: Band intensity of the proteins measured by the ImageJ software

β-Actin	Intensity			
	Control	MPP+	MET	MET+MPP+
n1	25.4372	33.671	20.2864	33.671
n2	27.5912	33.671	19.4164	25.155
n3	24.155	30.467	17.5104	30.467

TIMM23	Intensity				TIMM23/β-Actin				Relative to Control			
	Control	MPP+	MET	MET+MPP+	Con-trol	MPP+	MET	MET+MPP+	Control	MPP+	MET	MET+MPP+
n1	33.3322	19.084	39.6952	8.121	1.310372	0.566779	1.956739	0.241187	1	0.432532	1.49327	0.18406
n2	33.5912	19.875	31.6624	6.6244	1.217461	0.590271	1.630704	0.263343	1	0.484837	1.339431	0.216305
n3	32.9832	17.312	33.5912	5.0014	1.365481	0.568221	1.918357	0.164158	1	0.416133	1.404894	0.12022

NDUFS3	Intensity				NDUFS3/β-Actin				Relative to Control			
	Control	MPP+	MET	MET+MPP+	Con-trol	MPP+	MET	MET+MPP+	Control	MPP+	MET	MET+MPP+
n1	24.1612	17.8904	21.5703	10.5435	0.949837	0.53133	1.063289	0.313133	1	0.55939	1.119443	0.32967
n2	24.2162	18.7164	17.8824	9.8844	0.877678	0.555861	0.920995	0.39294	1	0.633331	1.049353	0.447704
n3	25.4052	20.822	19.6512	11.972	1.051757	0.683428	1.122259	0.39295	1	0.649796	1.067032	0.373613

TIMM23, Translocase of Inner Mitochondrial Membrane 23
NDUFS3, NADH Dehydrogenase (Ubiquinone) Fe-S Protein 3
MPP^+, 1-methyl-4-phenylpyridinium
MET, metformin
n, number of experiment
(Raw data to Figure 3, main document)

EXCLI Journal 2019;18:812-823 – ISSN 1611-2156
Received: August 05, 2019, accepted: September 03, 2019, published: September 10, 2019

Supplementary Table 4: The mitochondrial membrane potential was examined using the fluorescent JC-10 probe, and analyzed with a flow cytometer. Q1 (quadrant 1) indicates cells emitting light of the JC-10 monomer form, and Q4 (quadrant 4) indicates cells emitting light of the JC-10 aggregated form.

Control	Q1	Q2	Q3	Q4	sum
n1	59.9	29.3	9.1	1.7	100
n2	78.5	11.5	8.8	1.2	100
n3	61	28.8	9.1	1.1	100
mean	66.46667	23.2	9	1.333333	
MPP^+	Q1	Q2	Q3	Q4	sums
n1	2.9	2.8	41.3	53	100
n2	2.45	3.25	43.6	50.7	100
n3	2.59	2.61	41.6	53.2	100
mean	2.646667	2.886667	42.16667	52.3	
MET	Q1	Q2	Q3	Q4	sum
n1	56.4	3.3	40.3	0	100
n2	61.3	1.1	37.6	0	100
n3	50.8	3.2	46	0	100
mean	56.16667	2.533333	41.3	0	
MET+MPP^+	Q1	Q2	Q3	Q4	sum
n1	74.3	0.2	25.5	0	100
n2	58.8	1.1	40.1	0	100
n3	67.9	3.2	28.9	0	100
mean	67	1.5	31.5	0	

MPP^+, 1-methyl-4-phenylpyridinium
MET, metformin
n, number of experiment
(Raw data to Figure 5, main document)
